# Heatstroke-induced late-onset neurological deficits in mice caused by white matter demyelination, Purkinje cell degeneration, and synaptic impairment in the cerebellum

**DOI:** 10.1038/s41598-022-14849-9

**Published:** 2022-06-22

**Authors:** Kazuyuki Miyamoto, Motoyasu Nakamura, Hirokazu Ohtaki, Keisuke Suzuki, Hiroki Yamaga, Kaoru Yanagisawa, Atsuo Maeda, Masaharu Yagi, Munetaka Hayashi, Kazuho Honda, Kenji Dohi

**Affiliations:** 1 Department of Emergency, Critical Care and Disaster Medicine, Showa University School of Medicine, 1-5-8 Hatanodai, Shinagawa-ku, Tokyo, 142-8555 Japan; 2 Department of Anatomy, Showa University School of Medicine, 1-5-8 Hatanodai, Shinagawa-ku, Tokyo, 142-8555 Japan; 3 Department of Emergency and Disaster Medicine, Showa University Northern Yokohama Hospital, 35-1 Chigasaki-chuo Tsuzuki-ku, Yokohama, 224-8503 Japan; 4 Department of Functional Neurobiology, Tokyo University of Pharmacy and Life Science, School of Pharmacy, 1432-1 Horinouchi, Hachioji, Tokyo 192-0392 Japan

**Keywords:** Chronic inflammation, Disability, Neurodegeneration, Experimental models of disease, Fever

## Abstract

Global warming increases heatstroke incidence. After heatstroke, patients exhibit neurological symptoms, suggesting cerebellar damage. However, the potential long-term adverse outcomes are poorly understood. We studied the cerebellum after heatstroke in mouse heatstroke models. In this study, motor coordination disorder significantly appeared 3 weeks after heatstroke and gradually improved to some extent. Although white matter demyelination was detected at 1 and 3 weeks after heatstroke in the cerebellum, it was not found in the corpus callosum. The Purkinje cell numbers significantly decreased at 1, 3, and 9 weeks after heatstroke. The intensity of synaptophysin and postsynaptic density-95 temporarily appeared to attenuate at 3 weeks after heatstroke; however, both appeared to intensify at 9 weeks after heatstroke. Motor coordination loss occurred a few weeks after heatstroke and recovered to some extent. Late-onset motor impairment was suggested to be caused by cerebellar dysfunctions morphologically assessed by myelin staining of cerebellar white matter and immunostaining of Purkinje cells with pre- and postsynaptic markers. Purkinje cell number did not recover for 9 weeks; other factors, including motor coordination, partially recovered, probably by synaptic reconstruction, residual Purkinje cells, and other cerebellar white matter remyelination. These phenomena were associated with late-onset neurological deficits and recovery after heatstroke.

## Introduction

Heatstroke, a systemic disease caused by exposure to high ambient temperature (AT) and relative humidity (RH)^1^, is estimated to increase owing to the recent global warming^2,3^. Since patients with heatstroke often develop multiple organ damage in critical care, they are primarily treated with whole-body cooling, hemodialysis, and plasma exchange^4^. Although the acute effects after a heatstroke are well-recognized, potential long-term adverse outcomes are poorly understood. Notably, damage to the central nervous system (CNS) after a heatstroke has not been fully elucidated, despite being known to decrease the level of consciousness and function, which results in a condition similar to neuronal damage following a high fever caused by viral or bacterial infections and inflammation^5^. Approximately 25% of patients with heatstroke exhibit convalescent or long-term neurological deficits, including motor dysfunction and cognitive impairment.

Moreover, more than 70% of patients with neurological symptoms exhibit long-term cerebellar damage, most commonly presenting with adverse long-term neurological outcomes^6^. The cerebellum plays an important role in motor coordination/learning, sensory integration, and coordinate transformation. Cerebellar damage results in increased postural sway, hypermetric postural responses to perturbations and optokinetic stimuli, and postural responses that are poorly coordinated with volitional movement, which induces deficits in balance and walking^7^. After a heatstroke, patients who exhibit certain neurological symptoms—disorientation, wobbling (Supplementary Video S1), and vertigo—are bedridden, thereby preventing patients from returning to normal life, suggesting cerebellar damage. In addition to these neurological symptoms, several reports have revealed cerebellar abnormalities on head magnetic resonance imaging (MRI) in patients with heatstroke (Supplementary Fig. S1)^8,9^. Moreover, several interesting reports showed that neurological deficits appeared several weeks after heatstroke rather than immediately afterward^10,11^, and some cases improved with time^9^, while others are permanent. However, it is not well understood how these late-onset neurological deficits post heatstroke appear and, in some cases, improve over time. Therefore, we focused on the influence of heatstroke on the cerebellum using a mouse heatstroke model.

Few studies have examined the CNS using a heatstroke model. Most CNS studies performed on animals after heatstroke have evaluated heat-induced cerebral ischemic injury^12^, hypothalamic injury^13^, and alcohol influence on the central amygdaloid nucleus^14^. These studies did not consider RH, and other heatstroke studies that did not examine the CNS also disregarded the RH impact and created desert-like conditions^15–18^. However, summer in temperate or tropical zones has high AT and RH. Even if AT remains the same, a higher RH accounts for a higher wet-bulb globe temperature (WBGT), suggesting a higher risk of heatstroke. Therefore, we recently established a mouse heatstroke model that accounts for AT and RH while monitoring WBGT. Our animal model presents low mortality (< 20%, 4 days after heatstroke) and is considered suitable for evaluating long-term adverse outcomes^19^. In this study, we examined late-onset motor coordination and cerebellar damage for 9 weeks in mouse heatstroke models.

## Results

### Motor coordination disorder appeared 3 weeks after heatstroke

**Figure 1:**
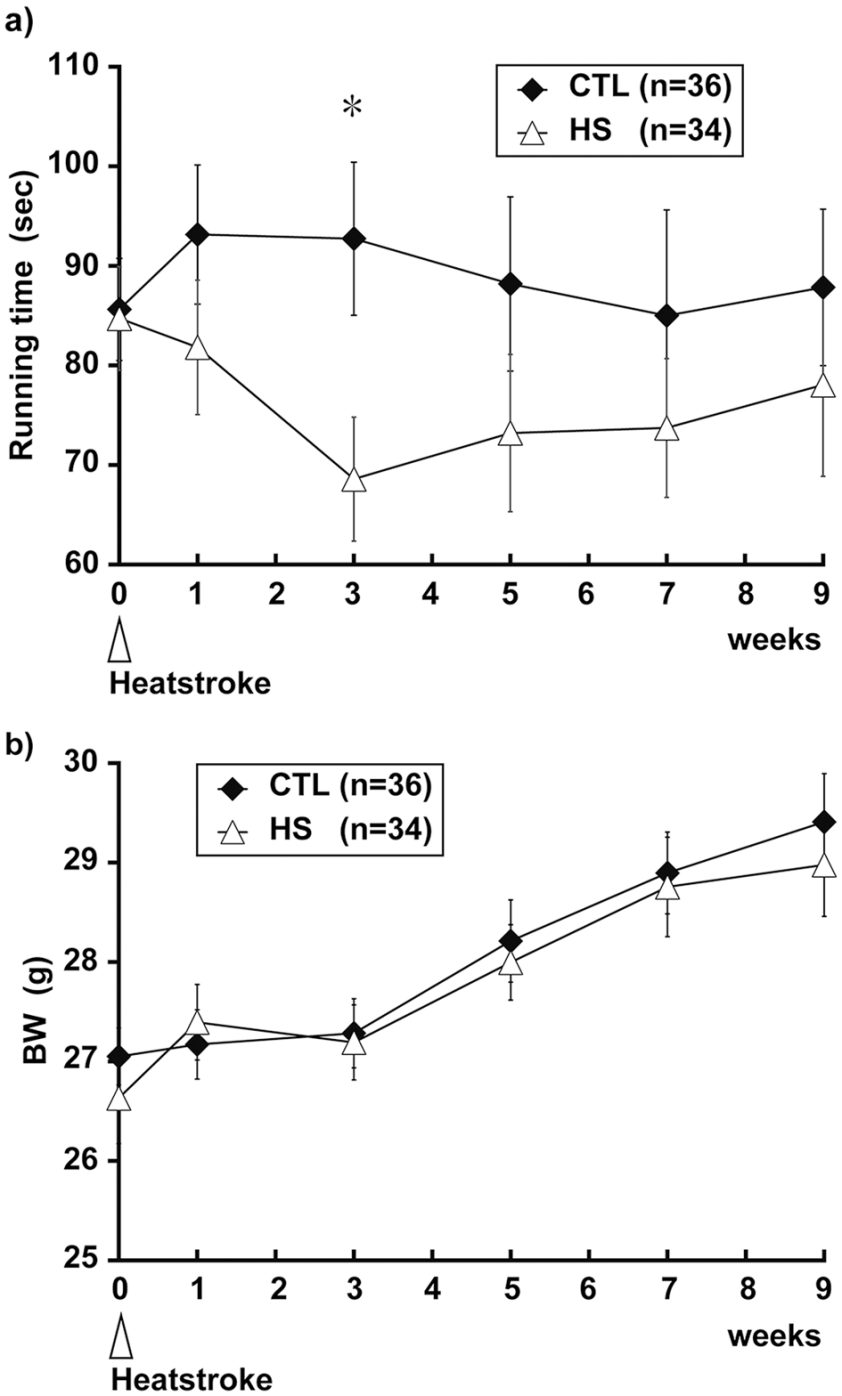
Motor coordination and body weight for 9 weeks after heatstroke. Running time of the rotarod test (**a**) and body weight (**b**, BW) in the control (CTL) and heatstroke (HS) animals. The running time of the rotarod test in the HS animals 3 weeks after heatstroke significantly decreased compared with that in CTL animals. BW in the HS and CTL groups did not change significantly over the experiments. Data are expressed as mean ± standard deviation (SD) (*P < 0.05, Tukey–Kramer test). *HS* heatstroke, *CTL* control, *BW* body weight.

Before heatstroke, the mean rotarod running time of mice in the control (CTL) and heatstroke (HS) groups was 85.6 ± 5.1 and 84.7 ± 5.2 s, respectively. Notably, 2 out of 36 mice in the HS group died within 1 day after heatstroke, and the remaining mice survived for 9 weeks. One week after heatstroke, the running time of the rotarod test in the HS group was 81.8 ± 6.8 s, whereas the running time in the CTL group was 93.1 ± 7.0 s. The running time of the rotarod test in the HS group 3 weeks after heatstroke further decreased to 68.8 ± 6.2 s and was significantly lower than that in the CTL group (92.7 ± 7.7 s, *P* < 0.05). The running time of the HS group gradually increased over time, whereas no differences were observed in the CTL group (Fig. 1a). To eliminate a feeding disorder after heatstroke, both groups of mice were weighed during the experiment, but these results were not statistically significant throughout the experiment (Fig. 1b).

### Demyelination detected at 1 and 3 weeks after heatstroke in the cerebellar white matter

**Figure 2:**
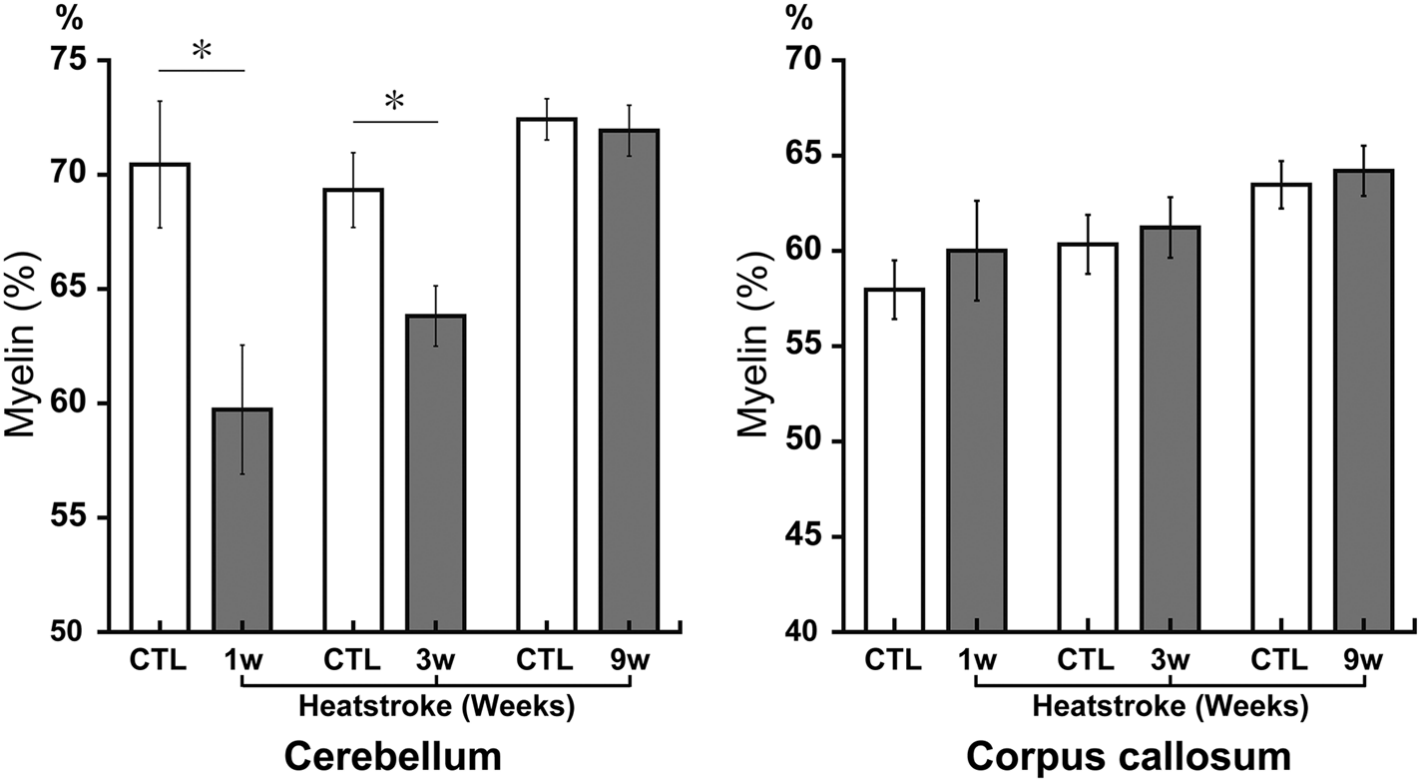
Cerebellum myelin sheaths in heatstroke animals temporarily decreased with Klüver–Barrera staining after heatstroke. The amount of myelination was semi-quantified in the white matter (WM) of the cerebellum and corpus callosum (CC) after heatstroke. Semi-quantification of the myelin staining revealed that the myelinated percentages were significantly decreased at 1 and 3 weeks after heatstroke, and it recovered 9 weeks later. No differences were observed between the HS and CTL groups in the CC. Data are expressed as mean ± SD (*P < 0.05, Student’s *t* test). *SD* standard deviation, *CTL* control.

The cerebellar white matter in the CTL mice was dense and homogeneous to fast blue. However, the white matter in the HS mice was heterogeneous and sponge-like. Semi-quantification of myelin showed that the myelin percentage was significantly decreased at 1 (CTL, 70.4% ± 2.8%; HS, 59.7% ± 2.8%; *P* < 0.05) and 3 weeks (CTL, 69.3% ± 1.6%; HS, 63.8% ± 1.3%; *P* < 0.05) after heatstroke (Fig. 2), suggesting demyelination. However, this decrease was not statistically significant among the groups at 9 weeks, suggesting recovery.

In contrast, we compared the myelin quantity in the cerebellum with that in the corpus callosum for the same sections to determine region specificity. Interestingly, we found no significant differences between the CTL and HS groups during the experiments.

### Purkinje cell number significantly decreased after heatstroke

**Figure 3:**
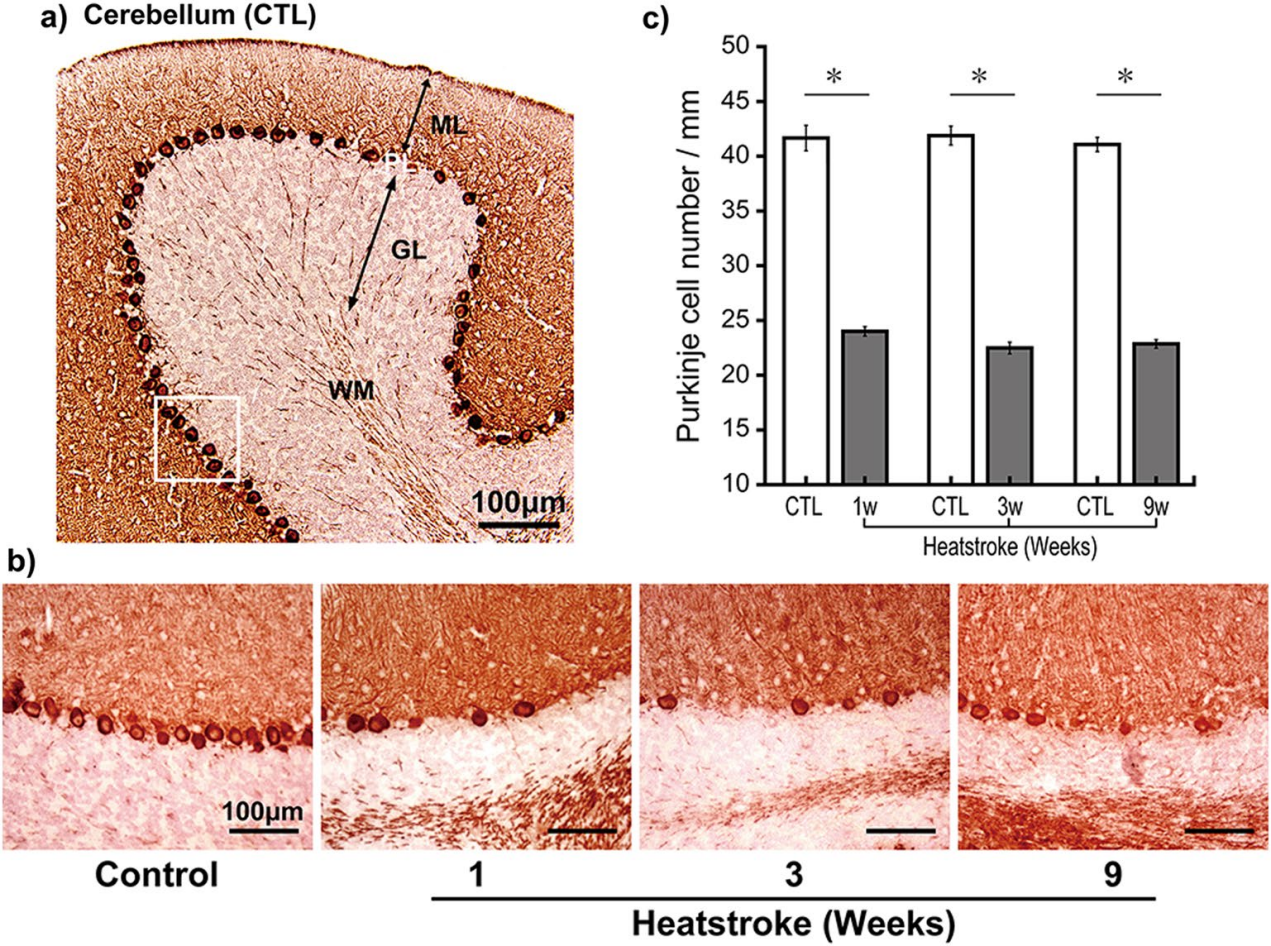
Purkinje cell number significantly decreased after heatstroke. To assess Purkinje cells, calbindin was immunostained in the brain and the calbindin-positive Purkinje cell numbers were counted. (**a**) Representative image of calbindin-immunoreactions in the cerebellum. Calbindin-immunoreactions stained the soma of Purkinje cells, the dendritic fibers that were widely stained in the molecular layer (ML), and the white matter. (**b**) Higher magnification images of the Purkinje cell layer in the control (CTL; left) group from 1 to 9 weeks after heatstroke. Purkinje cells in the CTL animals were aligned in an equidistant manner. However, the number of cells decreased after heatstroke. (**c**) The Purkinje cell numbers of the HS group were significantly decreased during the experimental periods after heatstroke (1, 3, and 9 weeks, n = 9, respectively) compared with the CTL group (n = 9). Data are expressed as means ± SD (*P < 0.05, Student’s *t* test). *ML* molecular layer, *PL* Purkinje cell layer, *GL* granular cell layer, *WM* white matter, *SD* standard deviation.

The Purkinje cells in the CTL mice aligned clearly in an equidistant manner (Fig. 3a). However, after heatstroke, the calbindin-positive Purkinje cells showed unequal distances and decreased numbers (Fig. 3b). The Purkinje cell number in the CTL versus HS groups was as follows: 1 week (41.7 ± 1.2 vs. 24.0 ± 0.4 cells/mm, *P* < 0.05), 3 weeks (41.9 ± 0.9 vs. 22.5 ± 0.5 cells/mm, *P* < 0.05), and 9 weeks (41.1 ± 0.7 vs. 22.9 ± 0.4 cells/mm, *P* < 0.05) after heatstroke (Fig. 3c).

### Expression of postsynaptic density-95 (PSD95) appeared to have temporarily attenuated at 3 weeks after heatstroke

**Figure 4:**
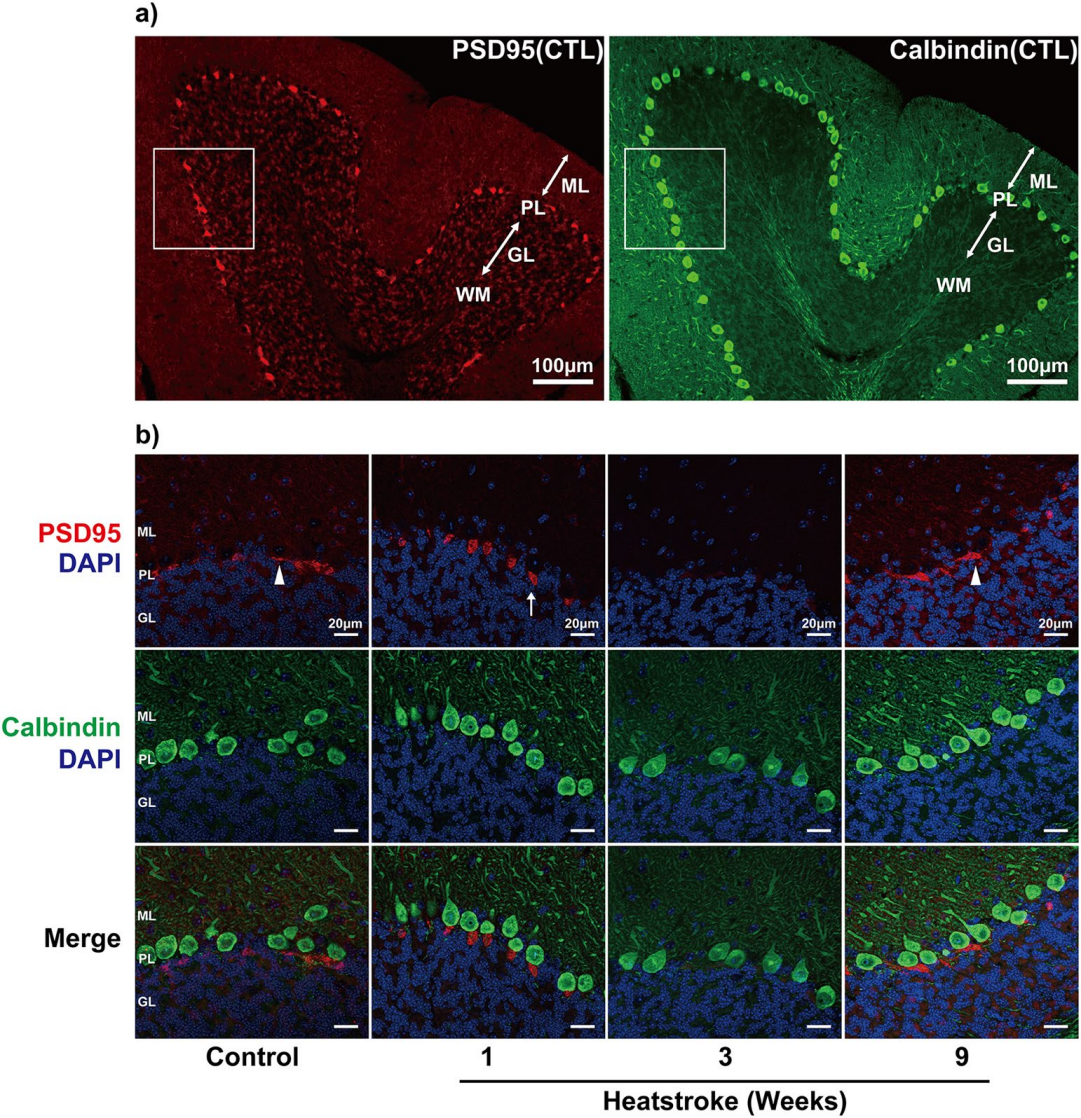
Immunostaining of postsynaptic density 95 in Purkinje cells after heatstroke. (**a**) Representative images of anti-postsynaptic density 95 (PSD95) (red), a postsynaptic marker, and calbindin (green) immunoreactions in the cerebellum in control mice. The PSD95 immunoreactions were observed in the Purkinje cells. Blue is due to 4′,6-diamidino-2-phenylindole nucleic staining. (**b**) The higher magnified images indicated that the PSD95-immunoreactions were located in the axon hillock of each Purkinje cell. The immunoreactions in the control group were widely recognized, such as plates, with relatively lower density in the Purkinje cells (arrowhead). The PSD95 immunoreactions 1 week after heatstroke (arrow) were gathered and concentrated in the axon hillock of the Purkinje cells like pen points. The immunoreactions appeared to have attenuated at 3 weeks and reappeared more heterogeneously at 9 weeks. *ML* molecular layer, *PL* Purkinje cell layer, *GL* granular cell layer, *WM* white matter, *CTL* control.

**Figure 5:**
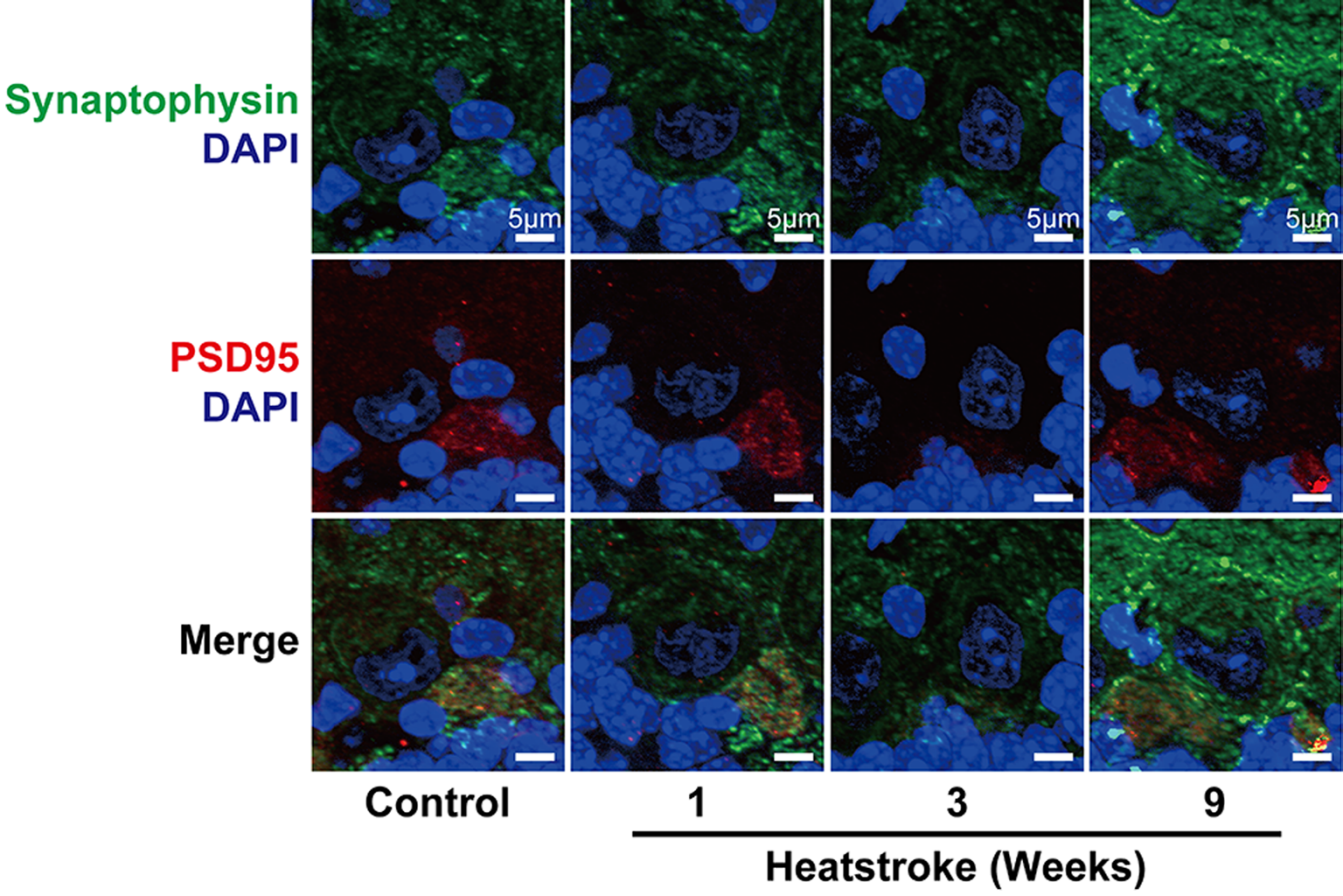
Expression of postsynaptic density-95 and synaptophysin around Purkinje cells. To estimate synaptic connections, pre- (synaptophysin, green) and postsynaptic (PSD95, red) markers were co-stained in the cerebellum and were observed with higher magnification images. Both postsynaptic density 95 and synaptophysin immunoreactions in the Purkinje cell layer appeared to have attenuated 3 weeks after heatstroke and intensified again after 9 weeks.

We examined Purkinje cell synaptic plasticity based on PSD95 expression. The distribution of PSD95-positive cells was strongly localized to the axon plexus. The PSD95-positive cells in the CTL group had a wide morphology, such as plates, but had low density. The PSD95-positive cells at 1 week after heatstroke were localized to the axon hillock of the Purkinje cells (looked similar to pen points). After 3 weeks, the immunoreactions appeared to have attenuated, although the calbindin-positive Purkinje cells were stained in the region. The positive reactions reappeared more heterogeneously at 9 weeks after heatstroke. Moreover, PSD95 immunoreactivity was observed again around the axon hillock of some residual Purkinje cells and appeared to have stronger intensities than that observed in the CTL group (Fig. 4). The co-staining of PSD95 with synaptophysin revealed that the intensity of synaptophysin immunoreactivity around the Purkinje cells appeared to have been the weakest at 3 weeks after heatstroke; however, both intensified at 9 weeks after heatstroke (Fig. 5).

## Discussion

It is estimated that the recent global warming will cause an increase in patients with heatstroke in the near future. However, there is poor understanding of the potential long-term adverse outcomes on the CNS, although patients often exhibit neurological deficits^8,20^.

In this study, we evaluated the neurological deficits using the rotarod test and brain histological evaluations (in the cerebellum) for 9 weeks using a mouse heatstroke model because disappearing and swelling of the Purkinje cells have been reported in autopsied brain tissues of patients with heatstroke^21^. Observation of brain diffusion-weighted MRI revealed hyperintensity in the bilateral cerebellar medullas in patients with heatstroke^22^. These reports indicate that cerebellar protection is a key therapeutic target in patients with heatstroke.

In our study, the running time of rotarod test was significantly shorter at 3 weeks after heatstroke and gradually returned to baseline at 9 weeks after heatstroke. These results suggest motor function/coordination impairment in our heatstroke model. In patients with multiple sclerosis, cerebellar ataxia is commonly observed, and diffuse white matter demyelination has been reported^23^. Therefore, we examined the morphology of the brain by Klüver–Barrera (KB) staining, but we could not recognize apparent cerebral infarction or hemorrhage. However, the cerebellum showed decreased intensity with KB staining and was recognized as sponge-like in the HS group. Semi-quantification of the KB-stained region revealed that the cerebellar white matter was significantly demyelinated between 1 and 3 weeks after heatstroke in the HS group. However, the intensity did not significantly differ from that of the CTL group at 9 weeks after heatstroke, suggesting remyelination. In contrast, demyelination could not be recognized in the corpus callosum during the experiments, and demyelination was cerebellar specific and might not have recovered until 9 weeks.

The calbindin-positive Purkinje cells in the CTL mice were aligned equidistantly on the Purkinje cell layer. The cell numbers had significantly decreased at 1 week after heatstroke and did not recover during the experimental periods, suggesting that the Purkinje cells degenerated. These results, including the rotarod test results, suggested that demyelination after heatstroke-induced cerebellar ataxia resulted in impaired motor coordination. These results resemble the outcomes observed in human patients with heatstroke^8,20–22^.

However, myelin recovered 9 weeks after heatstroke along with motor coordination, although the number of Purkinje cells did not recover during the experimental periods. To explain this discrepancy, we performed immunostaining of PSD95 and synaptophysin to examine synaptic plasticity and observed the Purkinje layer at a higher magnification. PSD95 was highly concentrated and tightly bound to the PSD of type 1 glutamatergic synapses, suggesting its critical role in protein assembly, synaptic development, and neural plasticity^23–25^. Synaptophysin is localized to the presynaptic vesicle membrane and contributes to membrane transport^26^. Its reduction has been considered to correlate with synaptic and neural impairments in Alzheimer’s disease and traumatic brain injury^27–30^. The PSD95 immunoreactions were recognized in the same direction of the axon plexus surrounding the axon hillock of each Purkinje cell as previously reported^31^ and were widely distributed, similar to plates or sheets, along with Purkinje cells. The degenerated Purkinje cells could not recover, and the cerebellar neuronal connections were temporally disrupted after heatstroke. However, the synapse and myelin staining results, including motor coordination, suggested that the surviving and residual Purkinje cells and the other cerebellar neurons were remyelinated and reconstructed new synaptic connections, probably inducing the recovery of partial motor coordination. It was suggested that these phenomena were associated with late-onset neurological deficits and recovery after heatstroke.

The cerebellum exhibits diversity and dynamism^32^, and cerebellar dysfunction improved to some extent with time in our mouse heatstroke model, although there is a case that showed a permanent neurological deficit in human heatstroke. Synaptic impairment might not be improved if the Purkinje cell damages were severe after heatstroke. Our results indicated there is a time before demyelination or synaptic impairment. Therefore, some therapeutic interventions during this period may contribute to improving neurological deficit after heatstroke.

In this study, we neither analyzed skeletal muscle impairment nor determined the spinal cord impairment and the peripheral nerve fiber innervation, because of a circulatory failure due to massive hemorrhagic shock and ischemia-induced motor neuron necrosis in the spinal cord’s ventral horn^33^. We did not systematically determine the presence of diffuse cerebral ischemia. Moreover, we did not perform electrophysiological examination and quantification of immunofluorescence staining (PSD95 and synaptophysin) to assess synaptic function after heatstroke. Further analysis is needed to clarify the heatstroke pathogeny and progression.

## Conclusions

In the present study, we determined the motor coordination loss within a few weeks after the onset of heatstroke. Motor impairment was suggested to be caused by cerebellar dysfunctions that were morphologically assessed by myelin staining of cerebellar white matter and immunostaining of Purkinje cells with pre- and postsynaptic markers. However, although the Purkinje cell number did not recover for 9 weeks, other factors, including motor coordination, were partially recovered, probably by synaptic reconstruction, residual Purkinje cell, and other cerebellar white matter remyelination. It was possible that these phenomena were associated with late-onset neurological deficits and recovery after heatstroke. Further research is required to clarify the mechanism of cerebellar dysfunction in heatstroke to establish new therapeutic strategies.

## Methods

Male C57/BL6J mice (age 10 weeks) were used in this study. All animals were purchased from SLC Japan Inc. (Shizuoka, Japan). The mice were allowed free access to food and water and were maintained on a 12-h light/dark cycle at room temperature (24 ± 2 °C) with constant humidity (40 ± 15%). All experimental procedures involving animals and clinical data were approved and overseen by the Institutional Animal Care and Use Committee of Showa University (#09022, 02003), which adhered to the ARRIVE guidelines. All methods were performed in accordance with the relevant guidelines and regulations.

Video of wobbling after human heatstroke and head MRI in patients with heatstroke were obtained from the participants. All experimental protocols were approved and overseen by the Clinical Trial Review Board of Showa University (F2019C83), which adhered to the CIOMS Ethical Guidelines for Biomedical Research. Informed consent was obtained from all participants and/or their legal guardians. Research has been performed in accordance with the principles of the Declaration of Helsinki.

### Protocol for heatstroke

The high AT and RH, resembling temperate/tropical summers, heatstroke model was used, which was based on our previous study^19^. A semi-enclosed heatstroke chamber (200 × 340 × 300 mm) made of acrylic was created by vertically stacking the animal cages in a greenhouse-like construction. An ultrasonic humidifier (USB-68, Sanwa, Japan) and a digital thermo-hygrometer (AD-5696; CA&D Company, Japan) were used for the humidification and monitoring of AT, RH, and WBGT. The heatstroke chamber was placed in an incubator (Bio-chamber, BCP-120F; TITEC, Japan) and preheated to the desired experimental temperature for ≥ 3 h. The humidifier was started 3 h before heatstroke to create a hot and humid environment. Meanwhile, the mice were allowed 3 h of water restriction, and the mildly dehydrated mice were placed in the heatstroke chamber and exposed to high AT (41 °C) and RH (> 99.0%) for 60 min. Subsequently, they were returned to the animal cage, where they could access food and water. Mice that were not heat-exposed were used as controls (CTL group).

### Behavioral study (rotarod test)

The rotarod test was performed according to a previous report^34^. A rotarod treadmill (Muromachi Kikai, Japan) consisted of a plastic rod (diameter, 3 cm; length, 10 cm) flanked by four large round plates (diameter, 57 cm). The rod rotates at a constant speed of 4 rpm at the beginning and continuously accelerates to a speed of 40 rpm for 5 min. The time each mouse spent on the rod was measured. Mice were trained with rotarod test once a week for five times before heatstroke. The behavior test in a mouse was performed twice within an interval of 5 min each and was expressed as the average of the trials. The animals were divided into two groups (HS and CTL) according to the scores of the last training (n = 36 in each group). The HS group mice were subjected to heat for 1 h. The CTL group (without HE) mice were prepared as a control. The HS and CTL groups were subjected to behavioral tests at 1, 3, 5, 7, and 9 weeks after heatstroke (Supplementary Fig. S2a).

### Tissue preparation

Under sodium pentobarbital (50 mg/kg, i.p.) anesthesia, the mice (HS, n = 9, per time course in each group) at 1, 3, and 9 weeks after heatstroke were transcardially perfused with 0.9% sodium chloride, followed by 10% neutralized formalin. The brain was removed and divided into two parts along the longitudinal cerebral fissure. Paraffin-embedded specimens of the right hemisphere of the brain were prepared. Subsequently, eight sagittal sections were sliced at a thickness of 5 μm at every 200 μm interval from the cerebral longitudinal fissure for histological examination, as described below. Age-matched CTL group mice were prepared to eliminate the influence of senescence (Supplementary Fig. S2b).

### Semi-quantification of cerebellar white matter demyelination

Cerebellar white matter demyelination was examined with the KB method without Nissl staining^33,35^. Eight sagittal sections of the cerebellar hemispheres were prepared at 200 µm intervals at the thickness 5 µm, stained with KB. These eight sections were tile scanned of the entire cerebellar hemisphere at 400 × magnification in each mouse using 9 animals per time course. The Luxol fast blue intensity, which stains myelin (fast blue) in the white matter, was then semi-quantified to evaluate demyelination. These areas were manually traced with blinded investigators and converted to black and white; black areas were semi-quantified using Scion Image for Windows (Scion Corporation, USA). The pixels of the black myelinated regions were divided by the pixels from the total traced areas and expressed as percentages. Eight serials of sagittal sections were averaged for each animal. These procedures were performed by two investigators (H.Y. and K.Y.) who were blinded to the experimental groups.

### Immunochemical staining and counting of Purkinje cells

Another serial series of eight sagittal sections were immunostained with antibodies against calbindin D-28k (calbindin) and used for counting Purkinje cells^36^. After removing the paraffin, using a series of xylene/alcohol solutions, the sections were incubated in 10 mM sodium citrate buffer (pH = 5.0) for 25 min to retrieve heat-induced antigen and immersed in 0.3% hydrogen peroxide/methanol for 30 min to block the endogenous peroxidase reaction. The sections were then incubated with a mouse Ig blocking reagent (M.O.M.; Vector, USA), followed by 5% goat serum wash to diminish the mouse endogenous immunoglobulin and non-specific reactions. The sections were then incubated overnight with a monoclonal mouse anti-calbindin antibody (1:2000; Swant, Switzerland) and incubated for 90 min with a biotinylated goat anti-mouse IgG the next day (1:200, DAKO, CA, USA). The immunoreactions were visualized by incubating with an avidin–biotin complex solution (Vector, USA) and diaminobenzidine (Sigma, USA). The calbindin-immunopositive Purkinje cells were determined and counted using the CellSens Standard software (Olympus, Japan). The number of Purkinje cells in the molecular layer of the cerebellum was counted twice manually in all lobules of the same section, and the average was calculated. Cell counting was repeated in the eight sagittal sections. This was also performed by an investigator (H.Y. and K.Y.) who was blinded to the experimental groups.

### Immunostaining of synaptic markers

Post- and presynaptic markers were co-stained in the cerebellum at 1, 3, and 9 weeks after heatstroke to estimate the synapse around Purkinje cells using four of the nine mice. After heat-induced antigen retrieval and blocking according to the protocol as mentioned above, the sections were incubated with either monoclonal rabbit anti-calbindin antibody (1:100, Cell Signaling, USA) or polyclonal rabbit anti-synaptophysin antibody (1:200, Proteintech, USA) with monoclonal mouse anti-postsynaptic density 95 (PSD95) antibody (1:400, BD, USA) overnight at 4 °C. After washing, the sections were incubated with Alexa 488-conjugated goat anti-rabbit IgG antibody (1:400; Thermo Fisher Scientific, USA) and Alexa 546-conjugated goat anti-mouse IgG antibody (1:400; Invitrogen, USA). Subsequently, cell nuclei were stained with 4,6-diamidine-2-phenylindole dihydrochloride (1:10,000; Roche, Germany) and incubated in 1.0 mM CuSO_4_ in 50 mM ammonium acetate buffer (pH = 5.0) to diminish autofluorescence^37,38^. Fluorescence was detected using an Axio Imager optical sectioning microscope with ApoTome II (Carl Zeiss, Germany). For control staining, the same steps were performed except incubation with primary antibodies.

### Statistical analysis

Data are reported as mean ± standard error of the mean. Student's *t* test was performed for comparisons between the two groups. A repeated measures analysis of variance and Tukey–Kramer test were performed for multiple comparisons. Statistical significance was set at a *P*-value of < 0.05.

### Ethics declarations

All experimental procedures involving animals were approved and overseen by the Institutional Animal Care and Use Committee of Showa University, which adhered to the ARRIVE guidelines. All human research protocols were approved and overseen by the Clinical Trial Review Board of Showa University, which adhered to the CIOMS Ethical Guidelines for Biomedical Research. Informed consent was obtained from all participants and/or their legal guardians. The research has been performed in accordance with the Declaration of Helsinki.

### Approval for animal experiments

All experimental procedures involving animals and clinical data were approved and overseen by the Institutional Animal Care and Use Committee of Showa University (#09022, 02003), which adhered to the ARRIVE guidelines.

### Approval for human experiments

All human research protocols were approved and overseen by the Clinical Trial Review Board of Showa University (F2019C83), which adhered to the CIOMS Ethical Guidelines for Biomedical Research. Informed consent was obtained from all participants and/or their legal guardians. Research has been performed in accordance with the principles of the Declaration of Helsinki.

## Supplementary Information

Supplementary Video S1.

Supplementary Information.

## Data Availability

The datasets used and analysed during the current study available from the corresponding author on reasonable request.
