# Supplementary material for: Heatstroke-induced late-onset neurological deficits in mice caused by white matter demyelination, Purkinje cell degeneration, and synaptic impairment in the cerebellum
Source: Sci Rep. 2022 Jun 22;12:10598. doi: 10.1038/s41598-022-14849-9 (PMC9217968; doi:10.1038/s41598-022-14849-9)
Supplement: Supplementary file 2 — Supplementary Information. [file 41598_2022_14849_MOESM2_ESM.pdf]

Supplementary Information

Heatstroke-induced late-onset neurological deficits in mice caused by white matter demyelination, Purkinje cell degeneration, and synaptic impairment in the cerebellum

Kazuyuki Miyamoto*, Motoyasu Nakamura, Hirokazu Ohtaki, Keisuke Suzuki, Hiroki Yamaga, Kaoru Yanagisawa, Atsuo Maeda, Masaharu Yagi, Munetaka Hayashi, Kazuho Honda, Kenji Dohi

Figure S1 Head magnetic resonance imaging (MRI) image of patients with heatstroke

Head MRI was performed 8 and 47 days after heatstroke. High signal intensity (arrow) was detected at the cerebellum on diffusion-weighted imaging 8 days afer heatstroke, which diminished over time.

Figure S2 Experimental design

Protocol of (a) behavior study (rotarod test) and (b) brain tissue sampling.

HS, heatstroke; CTL, control

Video S1

Tandem gait examination of the patient with heatstroke

Wobble during walk observed upon tandem gait examination.

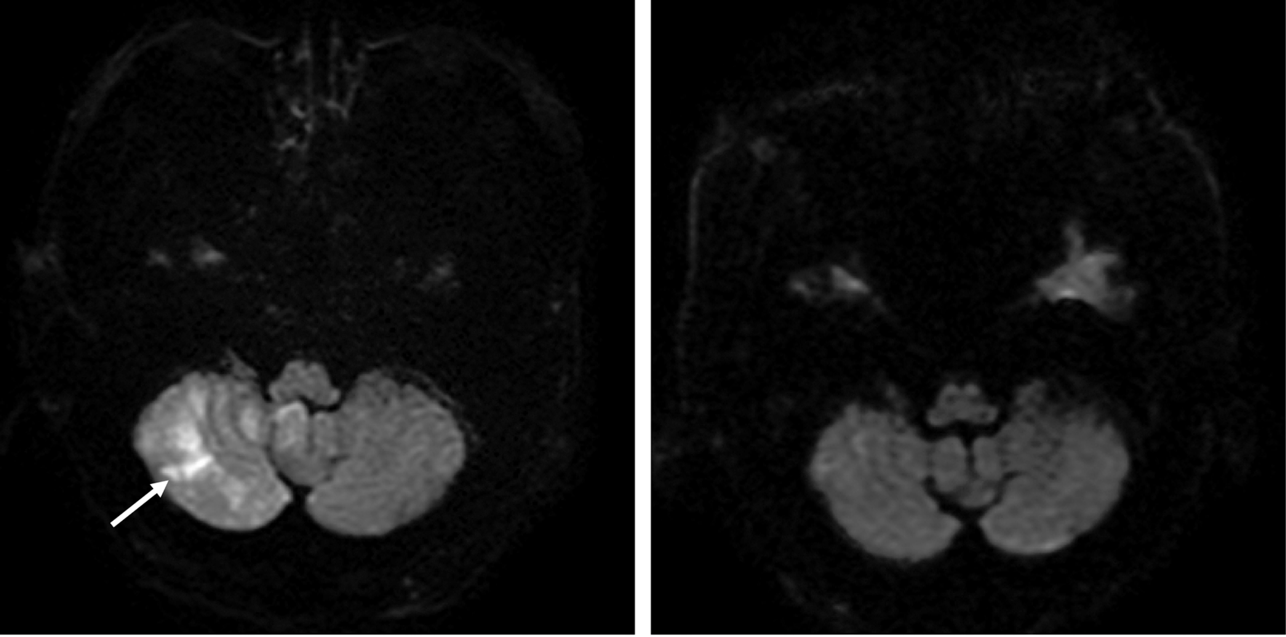

Day 8 Day 47

Supple Figure 1

Experimental design

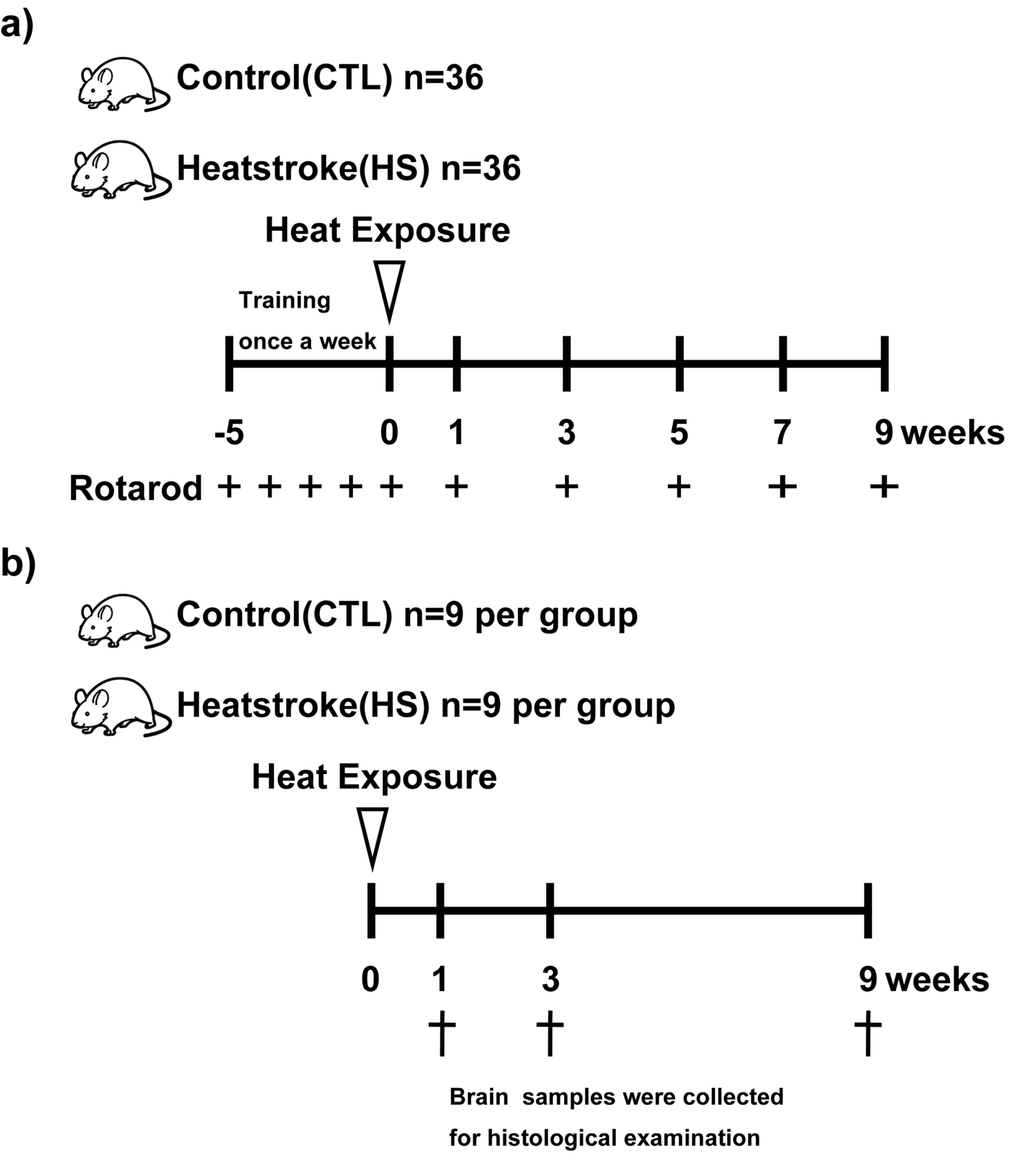

Supple Figure 2
